# Gender Identity Disorder and Schizophrenia: Neurodevelopmental Disorders with Common Causal Mechanisms?

**DOI:** 10.1155/2014/463757

**Published:** 2014-12-04

**Authors:** Ravi Philip Rajkumar

**Affiliations:** Department of Psychiatry, Jawaharlal Institute of Postgraduate Medical Education and Research (JIPMER), Dhanvantari Nagar, Pondicherry 605 006, India

## Abstract

Gender identity disorder (GID), recently renamed gender dysphoria (GD), is a rare condition characterized by an incongruity between gender identity and biological sex. Clinical evidence suggests that schizophrenia occurs in patients with GID at rates higher than in the general population and that patients with GID may have schizophrenia-like personality traits. Conversely, patients with schizophrenia may experience alterations in gender identity and gender role perception. Neurobiological research, including brain imaging and studies of finger length ratio and handedness, suggests that both these disorders are associated with altered cerebral sexual dimorphism and changes in cerebral lateralization. Various mechanisms, such as *Toxoplasma* infection, reduced levels of brain-derived neurotrophic factor (BDNF), early childhood adversity, and links with autism spectrum disorders, may account for some of this overlap. The implications of this association for further research are discussed.

## 1. Introduction

Gender identity disorder (GID) is a rare condition characterized by an incongruity between gender identity and biological sex. Individuals with this condition experience distress related to their biological sex and frequently express a desire to change it by hormonal or surgical means; in simple terms, they identify themselves as belonging to the opposite gender, and behave accordingly. GID is distinct from disorders of sexual development, such as chromosomal abnormalities or congenital adrenal hyperplasia, in that there is no anatomical or physiological ambiguity regarding the individual's sex; rather, there is a subjective dissatisfaction with it [1, 2]. The central role played by this dissatisfaction and distress has led to the condition being renamed “gender dysphoria” (GD) in the DSM-5, though the term “gender incongruence” (GI) has also been proposed by some authors [1].

Various theories have been proposed to account for GID/GD and related conditions, known as gender identity variants (GIVs) [3]. Abnormalities in genes related to gonadal hormone synthesis and action have been found in these individuals, suggesting that GID/GD is a disorder of sexual brain differentiation caused by exposure to altered hormone levels during a sensitive period [2, 3]; however, evidence for such a viewpoint has not been consistently found, and it fails to account for patients with late-onset GID who have few or no symptoms during childhood [4]. Alternately, GID has been conceptualized as a disorder of cortical representation of sex-specific bodily features, particularly the genitalia [5], but such a proposal does not account for the other behavioural changes seen in this condition. In this paper, I review theoretical and research evidence suggesting that GID is a neurodevelopmental disorder, involving the processes of brain lateralization and sexual differentiation, which is related to schizophrenia. First, the evidence of a symptomatic overlap between the two conditions is reviewed; next, evidence of common causal pathways and mechanisms is outlined and synthesized.

## 2. Clinical and Phenomenological Overlap between Schizophrenia and GID/GD

### 2.1. Comorbidity between the Two Disorders

A relationship between two distinct disorders can be inferred if they cooccur at a higher level than would be expected by chance. According to recent estimates, the prevalence of schizophrenia is approximately 1 to 8 per 1,000 population [6], and that of GID is even lower, estimated at around 1 in 10,000 for male-to-female GID and less than 1 in 25,000 for female-to-male GID [7]. Their cooccurrence would therefore be expected to be rare: a crude estimate, obtained by multiplying prevalences, would be less than 1 in 1,000,000 individuals.

Hospital and clinic-based studies of individuals with GID have found rates of schizophrenia far in excess of both this estimate and the general population prevalence; however, comorbid mental illness is likely to have been overrepresented in such samples. A Dutch survey of 186 psychiatrists evaluating patients with GID found that 31 (16.7%) reported seeing patients with comorbid GID and psychotic disorders, including schizophrenia [8]; however, it was not possible to calculate the percentage of patients with schizophrenia from the data provided by the authors, and the study has been criticized on the grounds of response bias, low external validity, and lack of a standardized means of confirming the diagnosis of psychosis [9]. A second Dutch study compared 20 patients who underwent gender-reassignment surgery and 27 in whom this treatment was deferred or delayed; no patient in the former group had schizophrenia, while 2 in the latter (7.4%) received this diagnosis, and a third was reported as having “psychotic episodes” [10]. An earlier clinic-based study obtained similar results; in a sample of 51 individuals with GID referred for psychiatric evaluation, 8% were found to have schizophrenia [11]. An unusual clustering was reported in African-American women with GID at a clinic in the United States; two of these five subjects (40%) were diagnosed with schizophrenia and a third was noted to have a “schizophrenic character” [12].

More modest but still significant results have been obtained in population-based samples. A recent study from Ireland found that 8 of 159 patients with male-to-female gender dysphoria (5%) had comorbid schizophrenia, as opposed to none of 59 patients with female-to-male GD [7]. Psychiatric evaluation of 230 self-referred applicants for gender-reassignment surgery in Spain, after excluding patients with psychosis but no clear diagnosis of GD/GID, identified six cases (2.6%) of psychosis, with equal rates in male and female subjects [13]. Though these figures are lower than those of the referral-based studies, they are likely to be closer to the true prevalence in this population and are still far higher than would be expected by chance alone. A study of Taiwanese students which measured symptoms, rather than diagnoses, found a strong correlation between symptoms of GID and schizophrenia in male students [14], also suggesting an effect of gender on this association.

Taken together, these studies provide indirect evidence for a link between schizophrenia and GID, on the grounds of their greater-than-chance cooccurrence. However, not all studies in adults or adolescents have been positive. A study of 579 subjects with GID from Japan found only one case of schizophrenia (0.17%) in a female-to-male patient, a rate which is comparable to the lower bound of general population values [15]; however, another publication involving the same sample makes it clear that five subjects with schizophrenia were excluded from the first paper, yielding a corrected prevalence of 1.02% [16]. A study of 435 patients attending a gender clinic in Texas found only four (0.92%) cases of schizophrenia [17]. In a sample of 83 Iranian patients with GID, no cases of schizophrenia were reported [18].

### 2.2. Beliefs Related to Gender Change in Schizophrenia

Beliefs or ideas related to gender change—historically termed* metamorphosis paranoica sexualis* by Richard von Krafft-Ebing—have been documented in schizophrenia for over a century. One of the most influential cases in the early literature on this disorder—Daniel Paul Schreber, whom Freud used as the basis for his psychodynamic formulations of schizophrenia—experienced bizarre delusions in which he believed that he would be transformed into a woman, and that this was part of his mission to redeem the world [19, 20]. Though no study has systematically examined the prevalence of such delusions in schizophrenia, there have been numerous case reports of patients with schizophrenia who had seemingly delusional beliefs related to gender identity [21–27]; in some of these cases, a weakening [21] or resolution [27] of gender dysphoria was reported after antipsychotic treatment, while in others, schizophrenic symptoms resolved and features of GID persisted, suggesting that they were distinct [26]. These reports suggest that there is a grey area between patients with schizophrenia, those with GID/GD, and those whose phenomenology does not clearly fit into either category. This situation is complicated by the fact that many researchers view the two diagnoses as mutually exclusive [15], which may lead to an underestimate of gender-related psychopathology in schizophrenia.

### 2.3. Gender Identity in Schizophrenia

Leaving aside overt delusions of gender change, are there subtler indications of gender disturbance in schizophrenia? A number of older studies have attempted to address this issue, though they are largely based on projective techniques of psychological assessment [28]. Evidence of disturbed gender role and gender identity in these studies includes distortions or omissions of anatomical features on being asked to draw human figures [29, 30], reduced satisfaction with body parts in male patients with schizophrenia [31], and inappropriate responses on psychological tests of masculinity-femininity [32, 33].

A more recent study of 90 patients with schizophrenia using the Bem Sex Role Inventory found that both men and women had culturally appropriate scores on the feminine role scale and low scores on the masculine role scale [34]. These findings provide evidence for a disturbed or distorted perception of gender identity and role in patients with schizophrenia; however, given the significant impact of culture on gender roles, they need to be replicated in non-Western settings.

### 2.4. Schizophrenia-Like Traits in Patients with GID/GD

In a similar way, some researchers have assessed schizophrenia-like traits or personality variables in individuals with gender identity disorder. Two studies have used the subscales of the Minnesota Multiphasic Personality Inventory (MMPI) to assess personality dimensions in subjects with GID. In the first, high scores on the femininity subscale were found in men applying for gender-reassignment surgery; however, an elevation in the schizophrenia subscale was found only in those men who were continuing to live as men [35]. In the second, female-to-male transsexuals receiving testosterone therapy as part of the management of GID showed a significant decrease in scores on the paranoia subscale of the MMPI-2 [36]. A study of Taiwanese university students found that gender dysphoria was strongly associated with schizoid personality in both men (OR 4.7; 95% CI 2.2–9.9) and women (OR 3.6; 95% CI 2.1–6.3) [37]. These findings suggest that schizophrenia-linked personality traits may be commoner in persons with GID, though gender and hormonal treatment may moderate this association.

## 3. Processes and Mechanisms Common to Schizophrenia and GID/GD

### 3.1. The Role of Prenatal Hormonal Factors in Schizophrenia and GID/GD

An influential model of GID, briefly alluded to above, posits that this condition is due to a disorder of sexual development which specifically involves the brain, but spares the internal and external genitalia [3]. This theory has gained some support from brain imaging studies in which sexually dimorphic brain regions resemble those of the desired rather than the anatomical gender in subjects with GID [38–40]. There is evidence that some brain structures may show a similar variation in schizophrenia, with men showing a “feminized” pattern and women showing a “masculinized” pattern [41, 42], leading to the proposal that schizophrenia itself may be related to gender-atypical brain development, perhaps caused by prenatal hormonal imbalances [43, 44]. However, there are important differences in brain structure across the two disorders: most importantly, the thickness of the cerebral cortex is increased in male-to-female transsexuals [45], while schizophrenia has been consistently associated with decreased cortical thickness, irrespective of gender [46–48]. This suggests that even if both disorders are related to common neurodevelopmental pathways, the changes seen in GID are less severe and more region-specific than those seen in schizophrenia.

Apart from the direct evidence of brain imaging studies, indirect evidence of prenatal hormonal influences can be obtained by studying the ratio of the length of the second and fourth digits (the 2D : 4D ratio) in adults; higher ratios indicate a more “feminized” pattern. In persons with GID, evidence of an altered 2D : 4D ratio has been reported in several studies [49–51], though some researchers have found a specific association only in men [49] or women [50, 51]. These alterations are generally in the direction of individual's self-identified sex, with female-to-male subjects showing a more “masculinized” pattern than control women, and male-to-female subjects showing a more “feminized” ratio. Very similar alterations in the 2D : 4D ratio have been reported in schizophrenia, more specifically in male patients where a reversal of normal sexual dimorphism has been documented [52, 53]. Similar abnormalities are also found in schizotypal disorder, a condition which is genetically and clinically related to schizophrenia [54, 55]. This alteration, especially in male patients with schizophrenia, has also been tentatively linked to abnormalities in prenatal testosterone exposure, a mechanism relevant to GID.

### 3.2. Cerebral Laterality and Handedness

Besides indicating sexual dimorphism, the finger length ratio is also an indicator of cerebral lateralization [56]. Alterations in cerebral lateralization may result in an excess of atypical hand dominance patterns, such as left-handedness or mixed laterality. A meta-analysis of 40 published studies found that schizophrenia was consistently associated with increased atypical hand dominance, particularly left-handedness [57]. Similarly, both male and female transsexuals have higher rates of non-right-handedness than healthy controls [58], and this has been documented even in young boys with childhood GID [59]. These findings suggest an altered pattern of lateralisation [58], presumably of developmental origin and at least partly linked to genes involved in neurodevelopment [60].

### 3.3. Toxoplasma Infection, Gender Identity, and Schizophrenia

Prenatal or childhood infection with the parasite* Toxoplasma gondii* has been identified as a risk factor for schizophrenia [61, 62]. Though evidence of this infection is not found in all patients with the disorder, it may be associated with a less favourable outcome [63] and with a higher risk of suicidal behaviour in younger patients with schizophrenia [64]. The exact mechanisms involved in this association are unknown but may include increased dopamine levels [61], alterations in brain development [65], or the activation of endogenous retroviruses [66]. Though there is no direct evidence linking* Toxoplasma* to GID, there is evidence suggesting that prenatal infection can result in “masculinization” of the foetus and even a predominance of male births in infected mothers [67]. In a follow-up study of seven adult patients with congenital toxoplasmosis, one male patient had developed a male-to-female GID and undergone gender-reassignment surgery [68]. It is therefore possible that* Toxoplasma* infection may be a common risk factor for both disorders, though this proposal needs to be tested serologically in individuals with GID. The link between* Toxoplasma* infection and suicidality is also not without relevance, as suicidal and self-injurious behaviour is very common in individuals with GID [15, 17].

### 3.4. The Role of Brain-Derived Neurotrophic Factor

Brain-derived neurotrophic factor (BDNF) is a nerve growth factor that plays a key role in brain development, as well as in the maintenance of neural plasticity in adult brains. The activity of BDNF can be influenced by several factors, including sex hormones such as oestrogen and testosterone [69]. A meta-analysis of studies conducted in patients with schizophrenia has identified a consistent, moderate decrease in blood BDNF levels compared to healthy controls [70]. A recent study has found evidence of decreased serum BDNF levels in patients with GID [71]. The authors of this paper have suggested that these changes may be due to childhood adversity [71] or stress related to the patients' “minority” status [72]. Yet, it is equally probable that low BDNF levels may signal a defect or deviation in normal brain development in this patient group [73], in the light of what is already known about the role of this molecule in the sexual differentiation of the brain [74]. Moreover, as discussed below, these two explanations are not mutually exclusive.

### 3.5. The Role of Childhood Attachment and Childhood Adversity

It is now well established that disturbances in parent-child attachment, including severe adverse experiences such as physical and sexual abuse, are associated with an elevated risk of schizophrenia [75]. A variety of biological and psychological mechanisms may underlie this association, including sensitization of dopamine pathways, impaired mentalizing abilities, and distortions of internal representations of the self and others; moreover, childhood adversity can itself alter brain development to some extent [76]. A relationship between disturbed childhood attachment and GID has been recognized for decades [77–81] and has unfortunately led to simplistic, reductionistic approaches to the management of this condition in some cases [82]. Nevertheless, more recent research has also found high rates (25%) of childhood maltreatment in male-to-female transsexuals; patients with a history of maltreatment had greater dissatisfaction with their body and worse mental health [83]. It is possible that, as in the case of schizophrenia, childhood maltreatment interacts with an underlying developmental vulnerability to lead to symptom formation in GID/GD; alternately, trauma-related release of stress hormones may lower BDNF levels [69], affecting childhood brain development.

### 3.6. Links between Autism, Schizophrenia, and GID/GD

Autism and related conditions, collectively known as autism spectrum disorders (ASD), are neurodevelopmental disorders which are distinct from schizophrenia but also overlap with it substantially on anatomical and neurocognitive grounds [84, 85]. The two conditions are also genetically linked; a family history of schizophrenia is a risk factor for ASD [86], and common genetic vulnerability loci have been identified [87]. Like schizophrenia, autism is linked to abnormalities in prenatal testosterone exposure, which is relevant to the pathogenesis of GID/GD [88, 89]. A study of individuals with ASD identified deviations in gender typicality, particularly lower overall levels of masculinity in both genders and higher levels of “tomboyism” in women with ASD [90]. Individuals of both biological sexes with ASD and comorbid GID/GD have also been reported in the literature [91, 92]. Conversely, studies of individuals with GID have identified high levels of autistic traits [93] and comorbid ASD [94, 95], though some of these results appear to be gender-specific. Deficits in empathising, a feature of ASD, are also associated with GD, especially in female-to-male subjects [96]. Though none of these studies have specifically examined links with schizophrenic symptoms or dimensions, they provide further indirect evidence for an etiological and phenomenological zone of contact between GID/GD and schizophrenia.

## 4. A Tentative Synthesis

Beginning with the observation that the comorbidity of schizophrenia and GID/GD is greater than would be expected, it is seen that patients with either condition may exhibit symptoms or traits of the other. Several converging lines of evidence, involving cerebral sexual dimorphism, laterality, prenatal infection, and childhood adversity, suggest that both these disorders have their roots in abnormal brain development and that their overlap may be explained by shared risk factors (such as* Toxoplasma* infection) or mechanisms (such as abnormal lateralization and sexual brain differentiation) via common biochemical pathways, such as prenatal hormonal imbalances or reduced BDNF expression and release. Schizophrenia is also associated with disturbances in body image and concept [97–99], which may be mediated through a frontal-limbic-temporal-parietal neural network [100]. Such processes would be relevant to an alternate model of GID, in which disturbances in body image representation, involving suppression or distortion of body maps in the parietal cortex, are considered to be the central feature [5, 101]. In either case, the existence of a link between the two disorders is certainly more than possible. Yet another intriguing possibility is raised by studies showing an association between GID and social cognitive deficits. It has been found by some researchers that individuals with female-to-male GID have deficits in empathising. This leads to difficulties in interacting with people of their own sex, who use empathising as a preferential mode of interaction [93, 96], and may cause them to identify more readily with males, who use “systemizing” more prominently than empathising [96]. Though this possibility was raised in the context of autistic traits, it may also be relevant to the schizoid or schizophrenia-like personality traits seen in individuals with GID/GD [14, 37] and provides a further pathway by which a neurodevelopmental vulnerability can lead to the development of this disorder.

## 5. Further Directions for Research

The proposal outlined above can be tested in several ways. Structural brain imaging studies can assess similarities and differences in sexually dimorphic structures in both groups and identify areas of overlap. Functional brain imaging and neuropsychological assessment may shed the light on anomalies of cerebral lateralization in these disorders. Clinical studies could focus on identifying body and gender disturbances in patients with schizophrenia and their first-degree relatives and schizotypal-like traits in patients with GID. The contribution of individual risk factors, such as* Toxoplasma* infection, can be assessed serologically. Genetic studies could examine the links between gender dysphoria, schizophrenia spectrum disorders, and related conditions such as autistic spectrum disorders. The consistency of, and correlations between, information obtained through these various methods would either clarify the link between schizophrenia and GID or refute it.

## 6. Limitations of the above Proposal

The model proposed above is not free of shortcomings. In the first place, it makes the assumption that schizophrenia and GID are both clear-cut syndromes, which is far from the case; schizophrenia is a heterogeneous disorder [102], and some of the clinical and developmental marker studies mentioned above have identified differences between male-to-female and female-to-male GID [7, 49–51]. Second, GID is comorbid with a variety of other psychiatric disorders, particularly mood and anxiety disorders, at rates higher than those reported for comorbid schizophrenia [7, 13, 16, 18]. It is not known if these disorders are the psychological consequence of living with GID, if they reflect shared vulnerabilities that need to be examined in their own right, or if patients with GID are at a nonspecifically elevated risk for a variety of disorders. Third, there are no systematic large-scale studies on gender disturbances in schizophrenia, or schizophrenia-like or schizotypal traits in GID, which would strengthen the case for the association proposed in this paper. Fourth, while the evidence presented earlier points towards a link between these two conditions, it does not explain the large differences between them. Fifth, owing to the relative rarity of comorbid GID and schizophrenia, no study has systematically examined the differences between patients with GID/GD alone, schizophrenia alone, and those with both conditions. Finally, there is evidence that risk factors for schizophrenia and GID/GD, apart from the ones discussed earlier, may be distinct. For example, factors such as migration, urbanicity, obstetric complications, cannabis use, and maternal viral infection are specifically linked to schizophrenia [6], but not to GID. Similarly, developmental antecedents of GID, such as early childhood cross-gender behaviour and same-sex sexual fantasies [103], are not specifically associated within schizophrenia. In the case of birth order, the relationship between GID and schizophrenia seems to be inverse; an earlier birth order is associated with schizophrenia in males [104], while a later birth order is associated with male-to-female GID [105, 106]. These divergences suggest that the two conditions may share certain causal pathways but do not overlap completely.

A further note of caution must be introduced here. The fact that scientific evidence suggests a link between GID and schizophrenia must not be taken to imply that GID is a psychotic disorder, that a wish for gender change is a form of schizophrenic thought disorder [107], or that such individuals must be treated with antipsychotic medication. It is beyond the scope of this paper to address the social and political controversies surrounding the diagnosis of GID [3, 108]. Research in this area should be driven by methodologically sound science rather than personal or political beliefs.

## 7. Conclusion

The available evidence, though limited, suggests that both gender identity disorder and schizophrenia are neurodevelopmental disorders and that they may share common causal mechanisms and risk factors. Further systematic investigation of these factors may provide a new perspective not only on these quite different conditions but also on the mechanisms and processes involved in normal brain development and sexual differentiation.
